# Gianotti-Crosti syndrome–like reaction in skin of color: An underreported sequela of molluscum contagiosum

**DOI:** 10.1016/j.jdcr.2024.04.024

**Published:** 2024-04-26

**Authors:** Landon K. Hobbs, Ali Rajabi-Estarabadi, Sophia Anagnostis, Carlos H. Nousari, Carlos Cohen

**Affiliations:** a Department of Dermatology, Broward Health Medical Center, Fort Lauderdale, Florida; b Nova Southeastern University College of Osteopathic Medicine, Fort Lauderdale, Florida

**Keywords:** Gianotti-Crosti syndrome, Gianotti-Crosti syndrome–like reaction, id reaction, molluscum contagiosum

## Introduction

Gianotti-Crosti syndrome (GCS) is a self-limited skin condition predominately occurring in young children and is presumed to be triggered by viral infections.^1^ Characteristic eruptions are monomorphous, erythematous papules, or papulovesicles with symmetric involvement over extensor extremities, face, and buttocks with sparing of the antecubital and popliteal fossae, soles, palms, and mucosal surfaces.^1^ GCS-like reaction (GCLR) is a similar condition that presents as a hypersensitivity reaction in response to molluscum contagiosum (MC). Although the rash closely resembles that of GCS, GCLR can be distinguished in that lesions are frequently accompanied by severe pruritus and localized over extensor surfaces of large joints in the presence of a concurrent MC infection.^2^

## Case report

**Fig 1:**
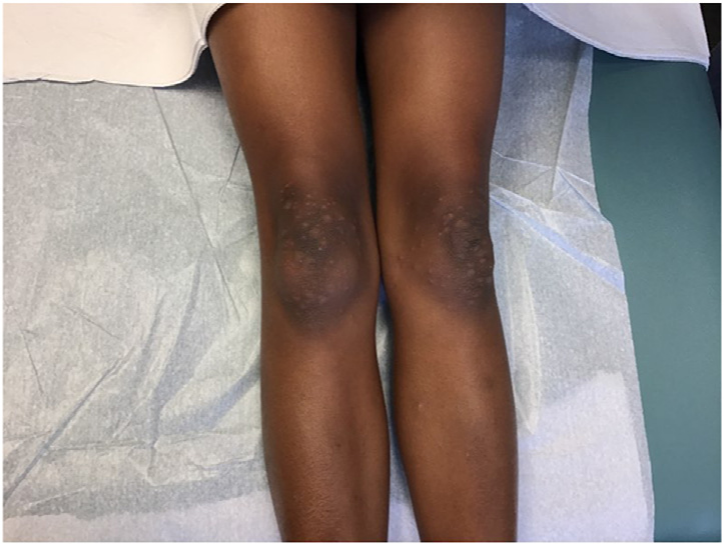
Symmetric distribution of erythematous papules and vesicles on extensor knees.

**Fig 2:**
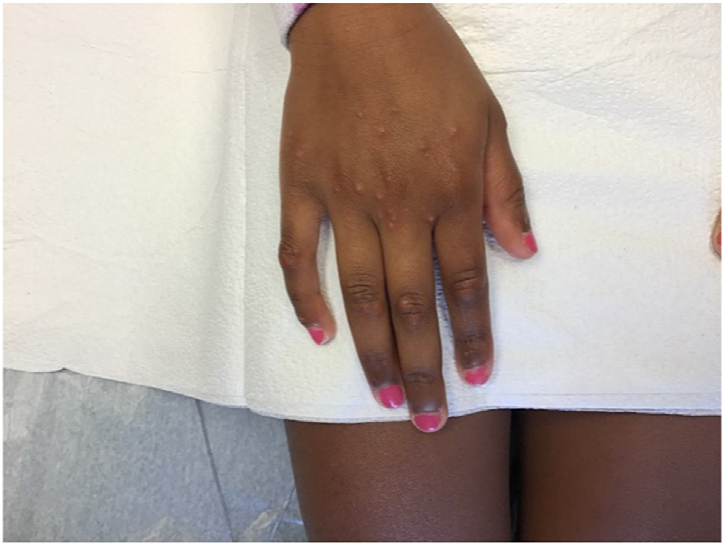
Erythematous papular and vesicular lesions on dorsal aspect of the hand.

**Fig 3:**
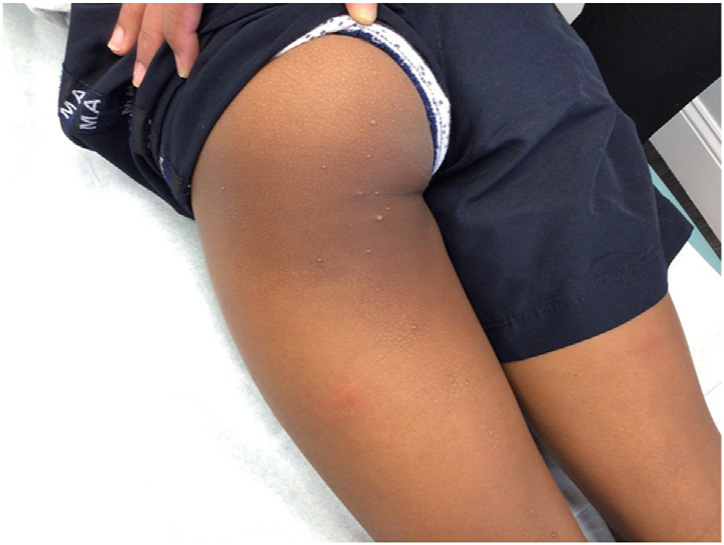
Few scattered and noninflamed molluscum contagiosum lesions 3 weeks prior to Gianotti-Crosti syndrome–like reaction eruption.

**Fig 4:**
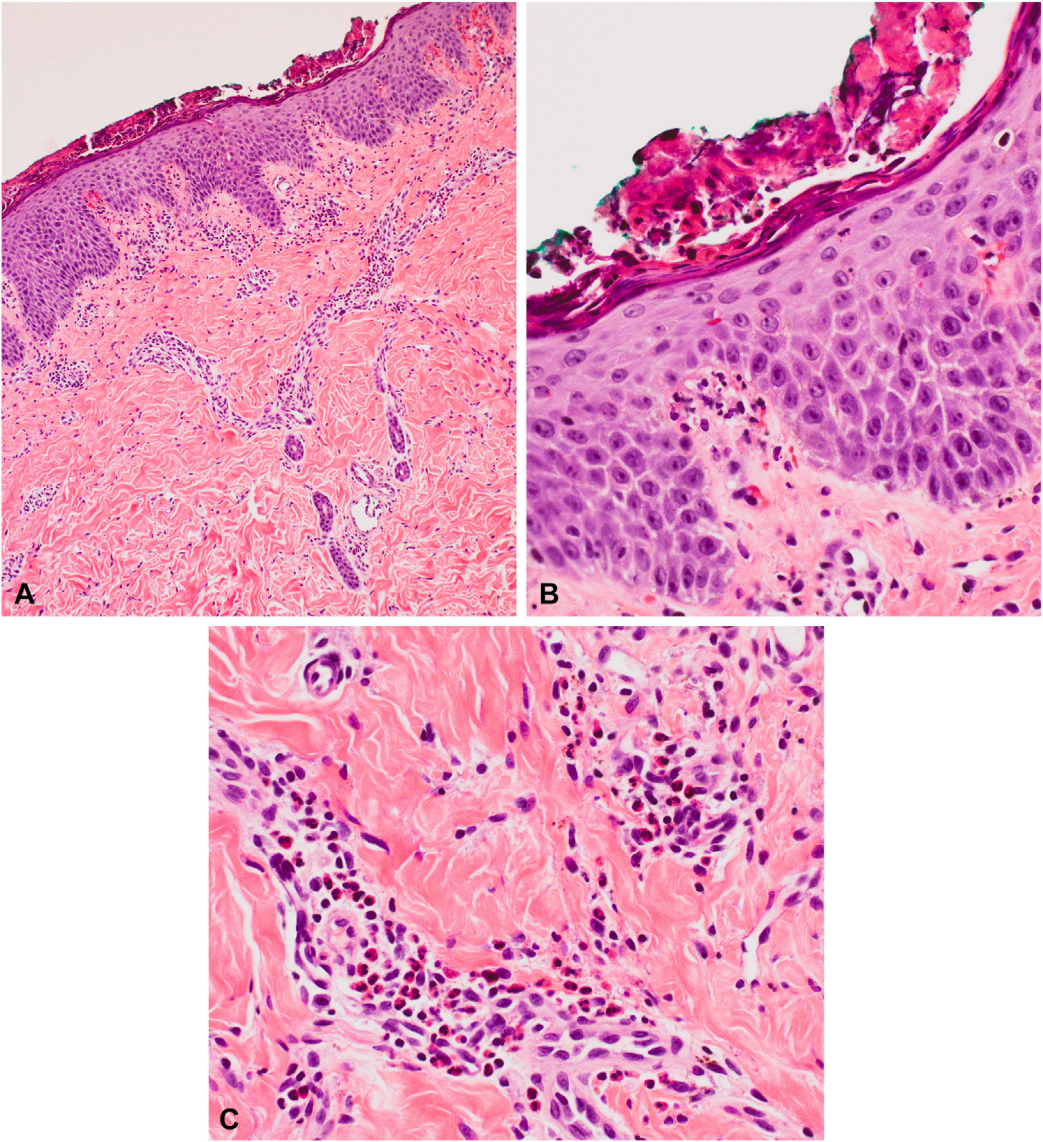
Papillary and upper reticular dermal, mostly perivascular, lymphocyte predominant polymorphous infiltrate with conspicuous eosinophils associated with spongiosis and irritational changes without acari or ectoparasite elements (**A-C,** Hematoxylin-eosin stain; original magnification: **A,** ×10; **B** and **C,** ×40. stain).

An 8-year-old Black girl with a history of eczema presented with a 1-week history of a moderately pruritic rash on the bilateral extremities. On examination, there were monomorphic pink and brown papules and vesicles on the extensor extremities, including bilateral knees, elbows, ankles, and hands without involvement of the face or trunk (Figs 1 and 2). There was no lymphadenopathy, fever, or other systemic symptoms. Serum studies were negative for cytomegalovirus, Epstein-Barr virus, and hepatitis B/C. Three weeks prior, the patient was diagnosed with noninflamed MC lesions localized to the left leg and buttock (Fig 3); 5 of which were treated with cantharidin at that time. The histologic examination of a punch biopsy of the skin lesion revealed papillary and upper reticular dermal, mostly perivascular, lymphocyte predominant polymorphous infiltrate with conspicuous eosinophils associated with spongiosis and irritational changes. Acari and ectoparasite elements were not identified (Fig 4).

Suspecting GCLR, the patient was treated with topical mometasone 0.1% with complete rash resolution at 5-week follow-up and no recurrence since.

## Discussion

MC is a self-limited viral skin infection caused by the MC virus and typically manifests as umbilicated papules.^2^ GCLR is a pruritic skin eruption thought to represent a cell-mediated hypersensitivity reaction, or possibly “id-like” reaction, to the MC virus.^2^, ^3^, ^4^ GCLR may indicate a good prognostic sign for MC as a dramatic reduction and complete clearance of MC lesions occurs after GCLR onset.^3^^,^^4^

GCLR is a clinical diagnosis; however, histopathology demonstrates nonspecific inflammatory findings of a superficial and mid-dermal perivascular infiltrate composed mainly of lymphocytes, eosinophils, and histiocytes, as well as spongiosis,^3^ as was seen in our patient. Laboratory tests are unnecessary, but ruling out viral infections such as cytomegalovirus, Epstein-Barr virus, or hepatitis B/C may be helpful in distinguishing GCLR from other conditions such as GCS.^1^^,^^2^

Though morphologically similar, it is important to distinguish classic GCS from GCLR in the setting of MC. GCS commonly presents as asymptomatic monomorphic, small papules on the cheeks and extensor surfaces of the extremities.^2^ In contrast, the lesions of GCLR are notably larger, intensely pruritic and can have varying morphology including papules/plaques, papulovesicles, and urticarial or target-like lesions.^2^^,^^3^ Clinicians should be aware that erythema seen in many conditions, including GCLR, may be subtle in darker skin types and appear more violaceous or hyperpigmented.^5^ In over 85% of GCLR cases in 2 reported case series, the upper and lower bilateral extremities were simultaneously affected, and involvement over large joints was a common finding.^2^^,^^3^ In our case, the eruption was localized primarily over the bilateral extensor knees with a few lesions over the dorsal aspect of the hand. GCLR, as in our case, typically involves large areas but it is not necessarily generalized like id reaction. Notably, the lesions appeared in areas that were not treated by cantharidin and in areas where molluscum was not present. Duration of GCLR and GCS varies with some series showing GCLR lasting 2 to 6 weeks and GCS lasting up to 10 weeks.^2^^,^^3^ In our case, the patient’s GCLR resolved within 5 weeks.

Additional diagnoses to consider in a patient with an inflammatory reaction in the setting of MC include inflamed MC lesions and molluscum dermatitis. Inflamed MC lesions typically present as erythematous, edematous papules, and papulonodules that may become pustular or fluctuant.^3^ In the study by Berger et al,^3^ inflamed MC lesions were observed in 22 (64.7%) of 34 patients with a GCLR compared with only 133 (20.1%) of 662 patients without a GCLR. It is important to clinically distinguish the papules/papulovesicles of a GCLR from inflamed MC lesions so that a true GCLR is not mistaken as a sudden increase in the number of molluscum lesions.^3^ Molluscum dermatitis presents as a pruritic eczematous eruption in the skin surrounding MC lesions.^3^ Berger et al^3^ observed no association between molluscum dermatitis and inflamed MC lesions or GCLR, suggesting the possibility of a different inflammatory pathway for noneczematous reactions such as inflamed MC and GCLR versus the eczematous reaction seen in molluscum dermatitis.

Prior to the diagnosis of GCLR, many patients diagnosed with MC undergo some form of treatment for the infection. In the study by Bürgler et al,^2^ two thirds of children received specific treatment for their MC infection before the onset of GCLR. These previous MC treatments included potassium hydroxide, cantharidin (as used in our patient) and curettage.^2^ One subject experienced the onset of GCLR 1 day after treatment with cantharidin.^2^ Our case reported the onset of GCLR within 2 weeks of treatment with cantharidin, and Berger et al^3^ reported onset of GCLR within 1 month of MC treatment in 38% of patients, suggesting that MC therapy may serve as a possible trigger for GCLR, indicating an area for further research.

Although GCLR is self-limited and usually resolves within weeks to months, lesions can be extremely uncomfortable for children. Thus, the management of GCLR should focus on patient/family education and reassurance as well as symptomatic treatment involving topical emollients, topical steroids and/or systemic steroids.^2^, ^3^, ^4^, ^5^, ^6^, ^7^, ^8^

Although sequelae resulting from a GCLR have not been reported, it should be noted that complications related to inflammation such as postinflammatory dyspigmentation are more likely to develop in patients with skin of color and manifest as hypopigmentation or hyperpigmentation.^5^ Hypopigmentation may be more noticeable in the darker skin of Black, Asian and Hispanic patients.^5^ Lesions caused by MC are usually benign and completely resolve; however, scratching at the lesions or mechanically removing the lesion can cause scarring and should be avoided, especially in skin of color.^8^

Given the exceedingly high prevalence of MC in children, it is important for clinicians to be aware of GCLR as a possible sequela. Yet, the scarcity of information available on the condition likely leads to underdiagnosis.^2^, ^3^, ^4^ This report serves to increase clinician awareness on GCLR and highlight its clinical presentation, emphasizing the appearance of characteristic lesions in skin of color and the possible complications for these patients.

## Conflicts of interest

None disclosed.
